# An Atypical Presentation of Rheumatoid Arthritis as an Asymmetrical Arthropathy

**DOI:** 10.7759/cureus.18452

**Published:** 2021-10-03

**Authors:** Marc Wong, Chong Jia Loon, C. Rajasoorya

**Affiliations:** 1 Internal Medicine, Sengkang General Hospital, Singapore, SGP

**Keywords:** asymmetrical, unilateral, radiculopathy, arthritis, rheumatoid

## Abstract

We report a rare entity of distinctly asymmetrical rheumatoid arthritis (RA) in a 71-year-old Chinese lady with a history of cervical radiculopathy secondary to trauma sustained during childhood. The joints on the side of the paresis were spared from severe clinical and radiological manifestations of RA. We review the plausible mechanisms that could explain the link between neurological impairment and rheumatoid joint involvement.

## Introduction

Rheumatoid arthritis (RA) is a chronic progressive inflammatory disease with the hallmark of this disease characterized by joint inflammation manifested by joint swelling, immune cell infiltrations, neovascularization, and synovial hyperplasia. Progression of this disease leads to bone erosion and joint destruction culminating in the classical presentation of symmetrical deforming polyarthropathy. In the case of our patient, asymmetrical RA changes were seen, attenuated in severity over her left hand that was concomitantly affected by long-standing paresis from lower cervical radiculopathy secondary to trauma sustained during childhood.

## Case presentation

A 71-year-old Chinese lady was seen for progressive bilateral lower limb weakness and prolonged right-hand pain. The lower limb weakness was predominantly proximal, worsened over three months, and had resulted in recurrent falls.

The pain in her right hand was described as intermittent, worse over the wrist and small joints with early morning stiffness lasting a few hours. She sought medical attention over the preceding one year and had been treated with multiple courses of prednisolone. She denied any symptoms of numbness and paresthesia of her extremities. Comparatively, her left hand was unaffected by joint pain or swelling. It was notably weaker and less functional since childhood after sustaining trauma.

She had no other associated rheumatologic manifestations such as alopecia, rash, photosensitivity, oral ulcer, dry mouth, or dry eyes. Her past medical history was significant for the lumbar vertebral body, L1 burst fracture requiring T11 to L3 pedicle screw construct.

**Figure 1:**
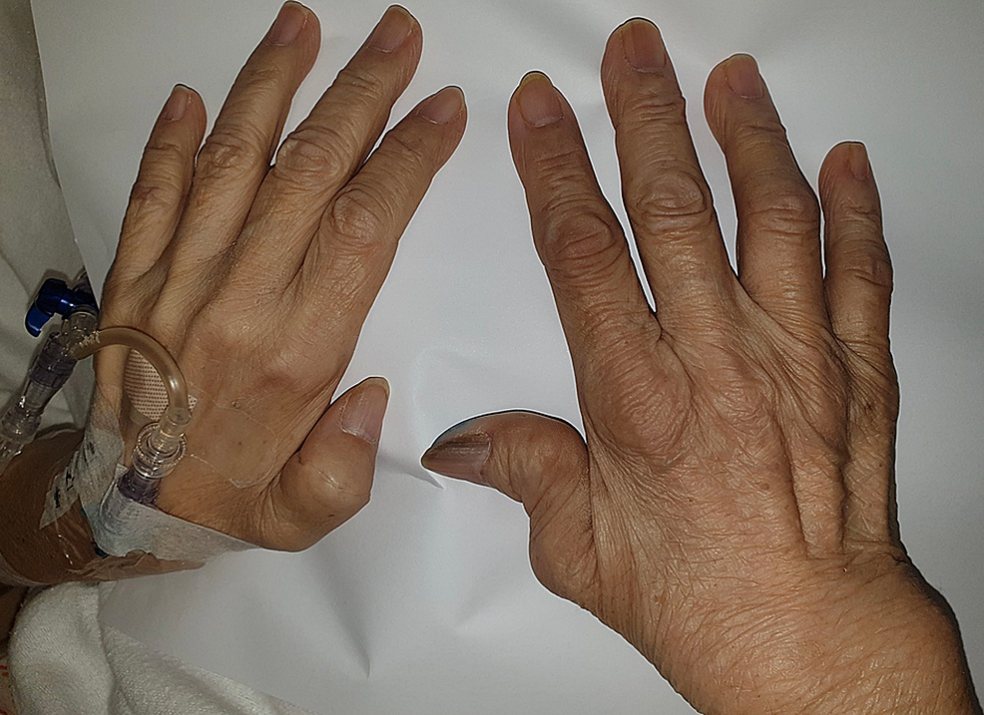
Bilateral hands with right hand showing ulnar deviation and Z thumb

**Figure 2:**
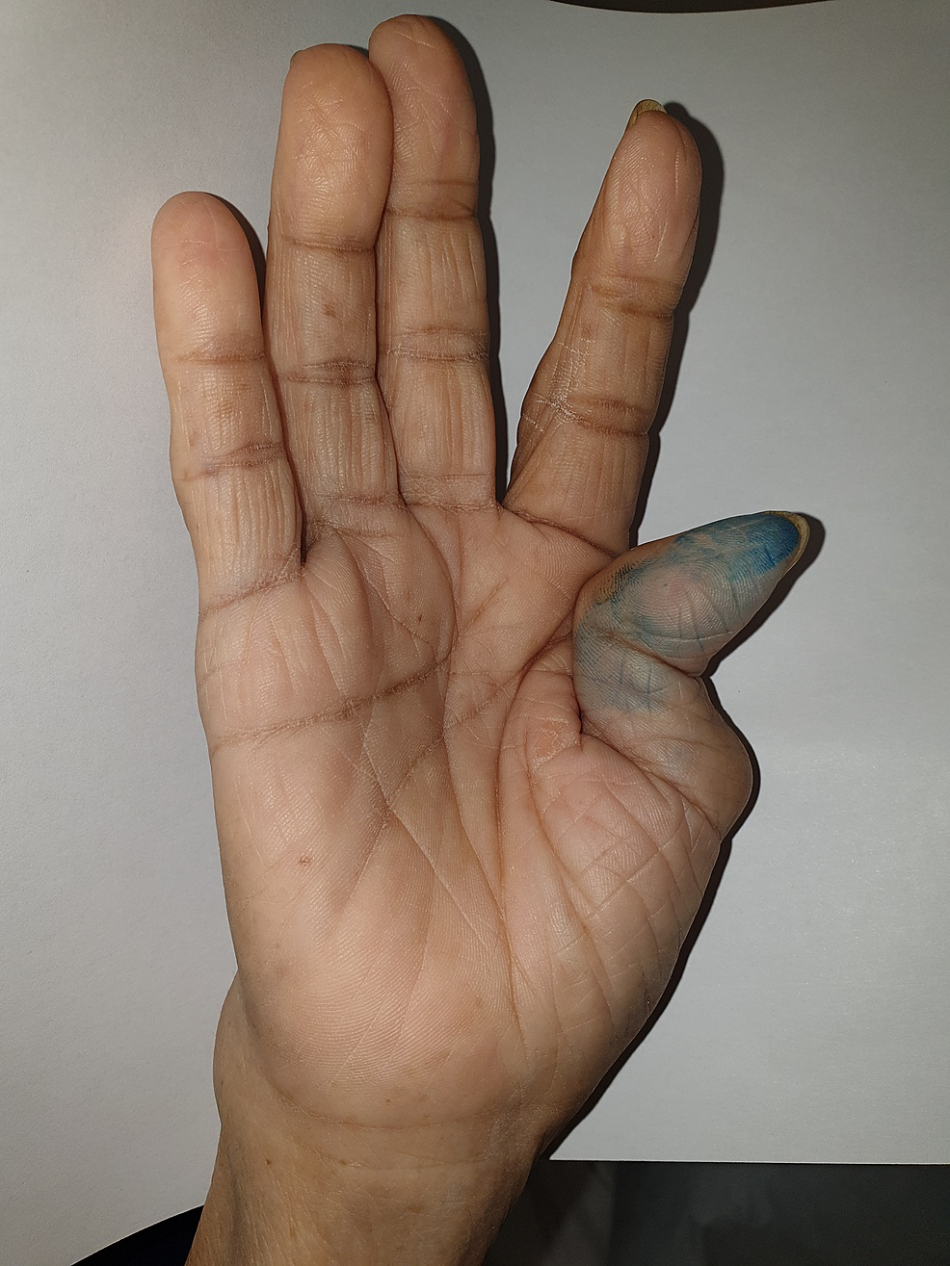
Right hand demonstrating Z thumb

Physical examination revealed asymmetrical bilateral upper limb polyarthropathy and proximal myopathy. Over her right hand, there was bony swelling over the wrist, ulnar deviation, and Z thumb deformity. There was swelling over the proximal interphalangeal joints (PIPJ) with tenderness over the second PIPJ (Figures 1, 2) with notable sparing of her distal interphalangeal (DIP) joints. She was not able to flex or extend her right wrist and was unable to make a fist with her right hand.

**Figure 3:**
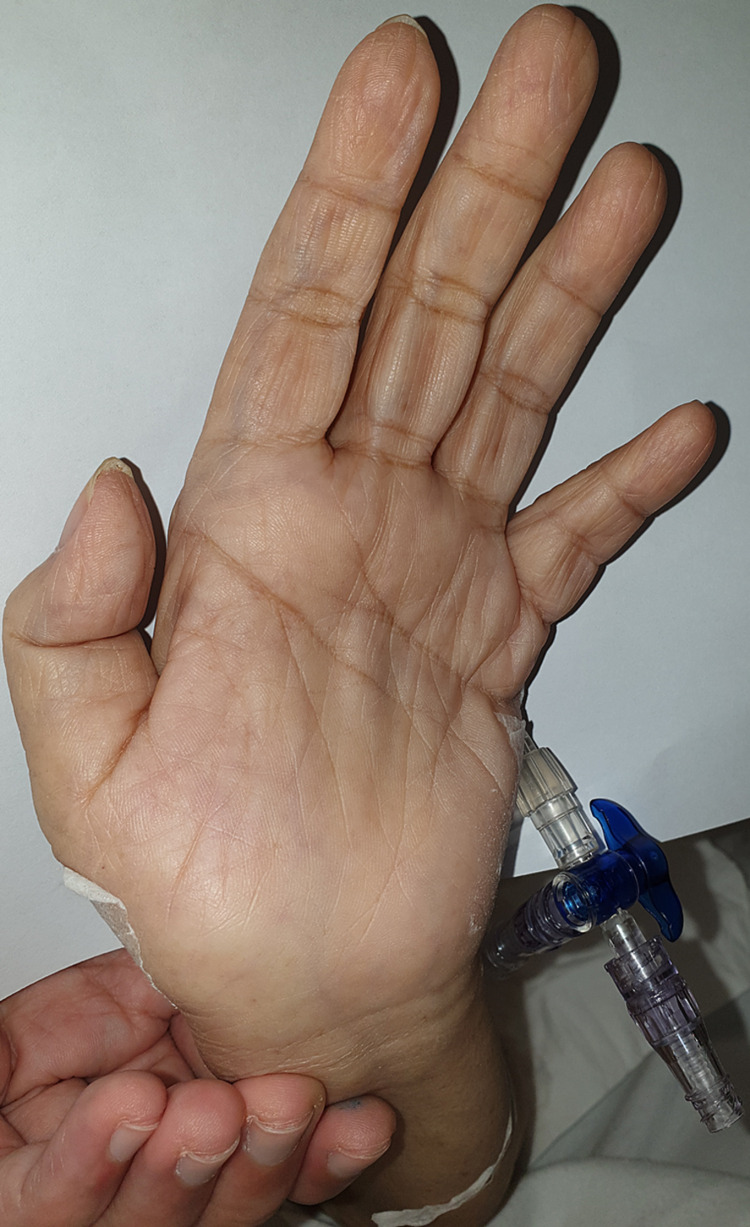
Left hand showing ulnar deviation

Over at her left hand, although there was an ulnar deviation, flexion and extension of the wrist were maintained up to 50 and 70 degrees, respectively (Figure 3). There was Boutonniere deformity of the left third digit with an examination of the other joints being unremarkable. She maintained the ability to make a fist with her left hand.

**Figure 4:**
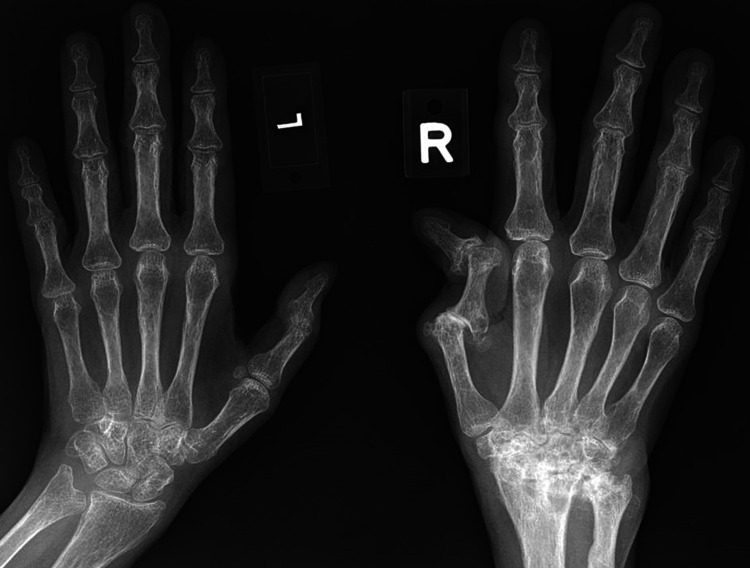
X-ray of the hands, PA view Advanced degeneration of the right wrist with the collapse of the carpal bones with boutonniere deformity of the right thumb while the left hand was unremarkable - save for osteopenia

**Figure 5:**
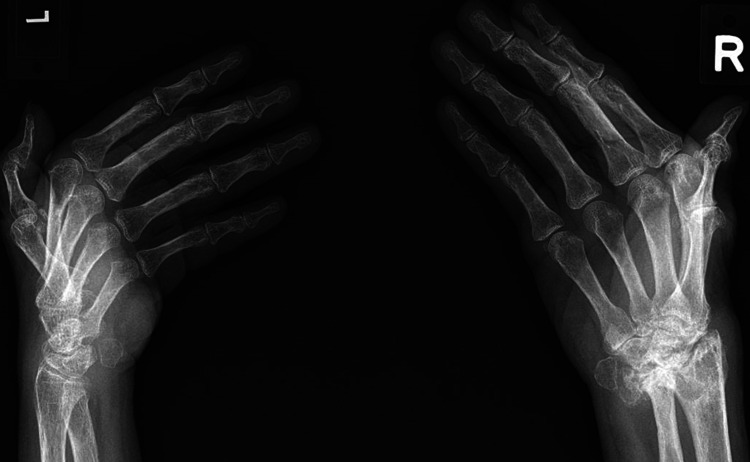
X-ray of the hands, ball catcher’s view

Investigations revealed elevated levels of rheumatoid factor (55 U/mL, normal range < 10.3 U/mL), anti-cyclic citrullinated peptide (12 U/mL, normal range < 5 U/mL), erythrocytes sedimentation rate (86 mm/hr, normal range 3-20 mm/hr), and C-reactive protein (65.6 mg/L, normal range 0.2-9.1 mg/L). A short Synacthen test revealed a sub-optimal response with basal levels of cortisol of 184 nmol/L rising to a maximal of 442 nmol/L at 60 mins after stimulation. X-ray of hands showed advanced degeneration of the right wrist with the collapse of the carpal bones, Boutonniere deformity of the right thumb, and bilateral juxta-articular osteopenia (Figures 4, 5). Nerve conduction study and electromyography studies reveal left lower cervical radiculopathy.

The patient's clinical presentation is compatible with seropositive RA with a European League Against Rheumatism (EULAR) score of 7 [1]. With prolonged use of oral steroids for arthralgia, she has now developed associated complications of osteoporosis-related compression fractures, proximal myopathy, and adrenal insufficiency. She was gradually tapered off steroids and had her function optimized through rehabilitation.

## Discussion

The classical presentation of rheumatoid hands is symmetrical deforming polyarthropathy. Since one of the earliest descriptions by Thompson and Bywaters in 1962 [2] of a patient with attenuated severity of RA in the affected limb after a stroke, there have been sporadic reports of such an entity as well as many hypotheses to explain the observed phenomenon. Glick [3] suggested that this asymmetry of decreased severity of RA joint changes was related to relative disuse. In that case report, a patient with monoparesis from poliomyelitis had attenuated RA joint deformities on the affected limb. Since then, similar reports have been few and far in between with the last notable one published in 1998 by Hiroshima et al. [4] on asymmetrical RA manifestations in the presence of monoparesis.

One commonly cited study in support of the idea behind the relation of attenuated RA joint changes and mechanical disuse is by Smith in 1978 [5]. In that study, it was proposed that resting is an effective treatment for RA. In the following year, Smith went on to propose that the reduction of movement protected against the local development of arthritis [6]. Critics of this study debated that the perceived benefits of rest therapy are largely seen in the DIP joints as seen in the degenerative process of osteoarthritis [4] and not in the PIPJ, which are classically affected in RA. At present, the exact mechanism of how disuse protects against RA remains uncertain. Since then there have been alternate hypotheses.

One important hypothesis postulates the involvement of the nervous system in inflammation. Since patients with neurological impairment had asymmetrical RA manifestations, could the nervous system itself be responsible for this asymmetry? Early studies peddled the idea that neuropeptides from the autonomic nervous system regulated the severity of inflammation in RA [7,8] but have not been proven since. In 2004, Keyszer et al. attempted to identify the responsible neuropeptide and studied various neuropeptides across paretic and non-paretic limbs. That study however failed to identify differing concentrations of neuropeptides between the paretic and non-paretic limbs of study subjects [9].

In 2014, Stangenberg et al. [10] used mice models and successfully demonstrated reproducible changes at the transcriptional level affected by denervation. In that study, they utilized the K/BxN T-cell receptor transgenic mouse model which allows an inflammatory phenotype to be induced by serum transfer, making it an ideal model to study RA [11,12].

These experimental K/BxN mice underwent surgical unilateral transaction of the sciatic and femoral nerve resulting in hemiplegia. For controls, a paired sham operation was performed on the contralateral hind limb. K/BxN serum was injected into the intraperitoneal space, inducing an inflammatory arthritis phenotype to facilitate the study of inflammation in the presence of denervation. This study demonstrated decreased inflammation in the joint spaces of the paws of mice in the presence of denervation.

Comparing the transcriptomes obtained from endothelial cells obtained from denervated paws and their controls revealed the downregulation of genes involved in the regulation of vascular permeability [10]. The downstream effects of this are postulated to be regulated through an effector molecule Junctional Adhesion Molecule B (JAM-B), which has previously been shown to have roles in the development of arthritis in mice models and endothelial cell stability [13].

JAM-B has a human homolog, JAM-C, known to be upregulated in the endothelial cells of the synovium affected by RA [13,14]. It facilitates leukocyte adhesion and transmigration to joint spaces as well as adhesion to synovial fibroblasts [15]. The cleaved product of JAM-C, soluble JAM-C is shed from endothelial surfaces in response to inflammation and functions as a pro-angiogenic mediator [15]. Neutralizing antibiotics targeted against JAM-C was also shown to delay the onset as well as to decrease the severity of inflammation of arthritis in mice models [16].

Taken together, it is postulated that innervation by the nervous system has a role in regulating the microvascular environment of inflammatory arthritis. It provides a signal required to maintain levels of effector molecules such as JAM-C, which in turn increases the vascular permeability of the microenvironment. When this signal is lost through denervation, it results in the alteration of the gene expression profile of endothelial cells in the synovium resulting in decreased vascular permeability. The decrease in vascular permeability could have then contributed to a reduction in the access to arthritogenic cells and molecules, resulting in a decreased inflammatory phenotype [10].

Currently, there is much work that remains to fully elucidate how the nervous system intricately regulates the immune system and its downstream effects other than to alter vascular permeability.

## Conclusions

The clinical manifestations of RA are attenuated in severity in the presence of paresis. While initially it was attributed to enforced immobility, increasing evidence points toward denervation leading to the downregulation of genes with a role in vascular permeability, conferring resistance to the development of arthritis. As paralysis can bring on venous and lymphatic stasis which may result in edema and increased likelihood of infections, a decrease in vascular permeability brought on by paralysis may help to minimize edema-related complications. Two important learning points can be gleaned from this case illustration, the first is the need to consider the possibility of a rare presentation of an asymmetrical RA with attenuation of its severity when there is a coexistent neurological impairment on the ipsilateral limb. Second, neurological impairment should not be missed in a patient presenting with asymmetrical arthropathy in a patient who otherwise has typical RA.

## References

[1] Aletaha D, Neogi T, Silman AJ (2010). 2010 Rheumatoid arthritis classification criteria: an American College of Rheumatology/European League Against Rheumatism collaborative initiative. Arthritis Rheum.

[2] Thompson M, Bywaters EG (1962). Unilateral rheumatoid arthritis following hemiplegia. Ann Rheum Dis.

[3] Glick EN (1967). Asymmetrical rheumatoid arthritis after poliomyelitis. Br Med J.

[4] Matsuno H, Tsuji H, Nakazawa F (1998). Asymmetrical manifestation of rheumatoid arthritis in poliomyelitis. Jpn J Rheumatol.

[5] Smith RD, Polley HF (1978). Rest therapy for rheumatoid arthritis. Mayo Clinic Proc.

[6] Smith RD (1979). Effect of hemiparesis on rheumatoid arthritis. Arthritis Rheum.

[7] Pattrick M, Doherty M, Dieppe P (1984). Unilateral exacerbation of rheumatoid arthritis by hemiparesis. Rheumatology.

[8] Decaris E, Guingamp C, Chat M (1999). Evidence for neurogenic transmission inducing degenerative cartilage damage distant from local inflammation. Arthritis Rheumatol.

[9] Keyszer G, Langer T, Kornhuber M (2004). Neurovascular mechanisms as a possible cause of remission of rheumatoid arthritis in hemiparetic limbs. Ann Rheum Dis.

[10] Stangenberg L, Burzyn D, Binstadt BA (2014). Denervation protects limbs from inflammatory arthritis via an impact on the microvasculature. Proc Natl Acad Sci U S A.

[11] Kouskoff V, Korganow AS, Duchatelle V (1996). Organ-specific disease provoked by systemic autoimmunity. Cell.

[12] Monach PA, Mathis D, Benoist C (2008). The K/BxN arthritis model. Curr Protoc Immunol.

[13] Weber C, Fraemohs L, Dejana E (2007). The role of junctional adhesion molecules in vascular inflammation. Nature Rev Immunol.

[14] Rabquer BJ, Pakozdi A, Michel JE, Gujar BS, Haines GK 3rd, Imhof BA, Koch AE (2008). Junctional adhesion molecule C mediates leukocyte adhesion to rheumatoid arthritis synovium. Arthritis Rheum.

[15] Rabquer BJ, Amin MA, Teegala N (2010). Junctional adhesion molecule-C is a soluble mediator of angiogenesis. J Immunol.

[16] Palmer G, Busso N, Aurrand-Lions M (2007). Expression and function of junctional adhesion molecule-C in human and experimental arthritis. Arthritis Res Ther.

